# Measuring the Digital Skills of Catalan Health Care Professionals as a Key Step Toward a Strategic Training Plan: Digital Competence Test Validation Study

**DOI:** 10.2196/38347

**Published:** 2022-11-30

**Authors:** Elisenda Reixach, Erik Andrés, Josuè Sallent Ribes, Montserrat Gea-Sánchez, Alícia Àvila López, Bea Cruañas, Anna González Abad, Ricard Faura, Montse Guitert, Teresa Romeu, Eulàlia Hernández-Encuentra, Sandra Bravo-Ramirez, Francesc Saigí-Rubió

**Affiliations:** 1 Fundació TIC Salut i Social Generalitat de Catalunya Barcelona Spain; 2 Departament de Salut Generalitat de Catalunya Barcelona Spain; 3 Servei Català de Salut Barcelona Spain; 4 Servei d'Inclusió i Capacitació Digital Departament de la Vicepresidència i de Polítiques Digitals i Territori Generalitat de Catalunya Barcelona Spain; 5 Faculty of Psychology and Education Sciences Universitat Oberta de Catalunya Barcelona Spain; 6 Faculty of Health Sciences Universitat Oberta de Catalunya Barcelona Spain

**Keywords:** digital health, eHealth, digital competences, digital literacy, information and communication technology, ICT, training

## Abstract

**Background:**

Despite Catalonia being an advanced region in terms of digital health adoption, the “Forum for Professional Dialogue” identified the need to improve information and communication technology (ICT) competences as one of the present and future challenges for health care professionals (HPs).

**Objective:**

We aimed to validate the digital competence test developed ad hoc for this study and to measure the digital competence level of Catalan HPs to establish their current level as the baseline for designing a strategic training plan.

**Methods:**

An exploratory observational study was conducted based on a voluntary survey where sociodemographic, professional and digital tool knowledge, digital tool use, and training needs data were collected and based on the score obtained from a digital competence test developed ad hoc. The digital competence test consisted of 2 “real-life scenarios” with 7 and 11 questions.

**Results:**

In total, 803 HPs, of whom 612 (76.2%) were women, completed the survey between June 28 and July 16, 2021. Most participants self-rated their digital competence level as either intermediate (384/803, 47.8%) or basic (357/803, 44.5%). The mean score in the digital competence test was 22.6 (SD 4.3). Therefore, most participants displayed a basic level of digital competence. The internal consistency of the digital competence test was 0.66, and the discrimination index of all questions was ≥0.2 for all items except for 1 question.

**Conclusions:**

This exploratory study highlights the need to improve the digital competence of HPs working in Catalonia, with special effort being made to provide training according to the specific needs of the different HP profiles. The results have informed the Health Plan for Catalonia 2021-2025 and lay the foundations for the development and deployment of a framework program for the digital competences of HPs. The developed digital competence test shows acceptable consistency for the objective pursued, although improvements are needed to fine-tune its accuracy.

## Introduction

### Background

Digital health (eHealth) is changing the way prevention, diagnosis, treatment, and health monitoring are provided to patients [1,2], while allowing universal access to equal, qualified, and cost-effective health care [3-5]. However, unlocking the full potential of eHealth is only possible when all actors (health care professionals [HPs], patients, managers, and policy makers) are committed to accepting and adopting information and communication technologies (ICTs) as a different way of providing or receiving care. Embracing the digital culture and developing professionals’ digital skills or competences to support digital transformation in the health care sector is fundamental to achieving this objective [6], because poor digital health competence is a common perceived barrier to the implementation of eHealth services [7-11].

Catalonia (Northeast Spain) is one of the most advanced regions in terms of digital health adoption across Europe [8,12,13]. Despite this, in 2018, the “Forum for Professional Dialogue” identified “the need to improve competences in ICTs to advance in their use and in the design of remote healthcare services” as one of the 17 main present and future challenges for HPs [14]. The “Digital Skills for HPs (COMPDIG-Salut)” project arose with the aim of meeting this challenge by addressing three objectives: (1) defining a specific digital competence framework for HPs; (2) creating a specific evaluation and accreditation model for HPs; and (3) drawing up actions to train and qualify HPs in digital competences. Therefore, knowing the current digital competence level of Catalan HPs is an essential first step upon which to build and address all COMPDIG-Salut project goals [15,16].

Despite the availability of a wide variety of free self-efficacy, knowledge-based, and performance-based digital competence assessments [16-22], these instruments were found to be too long, unvalidated, or too specific to measure HPs’ digital literacy levels. In 2009, the Government of Catalonia launched the Accreditation of Competence in ICTs (ACTIC) certificate with the aim of assessing citizens’ digital competence [23] on the basis of 3 levels: ACTIC 1-basic, ACTIC 2-intermediate, and ACTIC 3-advanced. The competences evaluated by this accreditation have been updated over the years and are currently aligned with the European Digital Competence Framework [24]. Currently, the attainment of the ACTIC 2-intermediate level certificate is a way of improving the professional development of HPs and the employability of graduates. However, the ACTIC intermediate certificate requires too much time and too many resources to complete; therefore, it was deemed unsuitable for the purposes of our research. Therefore, to assess the digital competences of HPs working in Catalonia, a digital competence test based on the ACTIC 2-intermediate certificate was developed ad hoc.

### Objective

The objective of this work is two-fold: (1) to validate the digital competence test developed ad hoc, which combines both skills and self-assessment, and (2) to assess the current digital competence level of HPs working in Catalonia and to identify the areas needing improvement.

## Methods

### Study Design

An exploratory, observational study based on a web-based survey was conducted by Fundació TIC Salut i Social between June 28 and July 16, 2021, among HPs currently working in Catalonia. By law [25], the definition of HPs, who make up the study population, includes dentists, dental hygienists, dental technicians, dietitians-nutritionists, occupational therapists, nurses, opticians or optometrists, pharmacists, physicians, physiotherapists, podiatrists, speech therapists, and other health or clinical specialists such as biologists, physicists or chemists, and psychologists. According to the last available report (2017) [14], the HP population in Catalonia consisted of 121,039 professionals working for public and private health care providers. Participation was both voluntary and anonymous.

The survey was conducted, and the results were reported in accordance with “Good Practice in the conduct and reporting of survey research” where appropriate [26] and in compliance with the General Data Protection Regulation.

### Survey Generation and Distribution

The survey included a section recording the participants’ characteristics (“descriptive survey”) and an objective digital competence test (Multimedia Appendix 1). The first section recorded demographic characteristics such as gender, age, professional profile, ownership of workplace (public or private [subsidized or nonsubsidized]), region, level of health care, professional experience, self-perception of digital competence, the use of digital tools for professional purposes, the need for training in digital tools for professional purposes, interest in receiving such training for personal purposes, and whether the participant had the ACTIC 2-intermediate certificate or an equivalent qualification.

The second section was the objective digital competence test. The ACTIC 2-intermediate level certificate served as the framework of reference to evaluate the digital competence level of HPs. The most appropriate and relevant digital competences for HPs were selected from those defined in ACTIC [27] to adapt the test to their reality and context (Table 1). Then, the indicators for each of these competences were selected to determine, in items, what aspects to evaluate. The indicators are observable characteristics and consist of specific tests, be they predefined measures or other types of qualitative information. After selecting the indicators, questions referring to them were formulated. These questions are related to the definition of observable behaviors that may be put into practice in different professional areas in the Catalan health care context. Observable behaviors are understood to be those practices or actions carried out by HPs during their professional activities (eg, searching for clinical information in databases, remote communication and collaboration with teams or patients, and using information management and content creation tools). As it was the achievement of competences that was being evaluated, the suitability of defining evaluation scenarios allowing respondents to be faced with challenges they needed to resolve was assessed. Attempting to resolve situations that are similar to real ones and giving the best digital response to the proposed challenges allows the degree of achievement of the indicators to be evaluated more effectively. It also enables other competences to be put into practice, such as problem-solving, critical thinking, and the analysis and responsible use of ICTs.

After formulating the questions for each indicator, it was necessary to close the loop by reviewing the entire process using a methodological process whereby the specification of new elements improves upon previous ones [28,29]. Test development followed an iterative process of expert consultation, prior pilot tests, and item review. For cross-validation, 8 members of the COMPDIG-Salut project were asked to evaluate the overall proposal and specifically whether the questions contemplated the defined indicators for the corresponding competence. The experts had to answer the survey questions and suggest any changes they deemed relevant by answering an open-ended question.

The digital competence test that we developed consisted of 2 “real-life” scenarios adapted to the health care sector with 7 and 11 self-developed questions. Each question had 4 possible answers, with a score ranging from 0 to 1 or 2, resulting in a total maximum score of 35. The final scores were classified into 3 levels: initial (2-9.9), basic (10-24.9), and intermediate (25-35). The survey took approximately 20 minutes to complete.

**Table 1:** Development of survey questions by competence.

Accreditation of Competence in Information and Communication Technologies competence and intermediate level indicator		Survey
**1.1 Searching for, selecting, and comparing information with digital tools**		
	The respondent uses advanced search parameters (language, update, publication date, region, Boolean operators, etc) using an assistant or different menus and options to optimize searches and readjust search criteria.	Case 1: Question 1
	The respondent is critical of the information and recognizes the limits of the internet as a single source.	Case 1: Question 2
**1.2 Organizing information and data with digital tools**		
	The respondent structures and classifies data coherently and accessibly using generic tools (spreadsheet or database) and specific tools (bookmark manager, contact manager, expense tracker, etc) to make searching easier.	Case 1: Question 6
**1.3 Analyzing, exploiting, and visualizing data with digital tools**		
	The respondent gathers data with digital tools (forms, surveys, etc) for specific objectives.	Case 1: Question 5
	The respondent uses and combines formulas and functions to perform simple operations.	Case 2: Question 7
**2.1 Interacting and sharing information and digital content**		
	The respondent acts as an example of communication for the rest of the digital community according to the context and the tool used.	Case 1: Question 4
**2.2 Collaborating with others via digital technologies**		
	The respondent identifies and uses digital tools, resources, and strategies to improve efficiency in the performance of tasks in collaboration with others.	Case 2: Question 1
	The respondent interacts by using the most appropriate (synchronous or asynchronous) communication tools and their advanced functions (user groups, broadcast groups, mailing groups, distribution lists, web-based meetings, etc) effectively.	Case 2: Questions 3 and 4
**3.1 Creating and publishing digital content**		
	The respondent selects and evaluates the most appropriate resources and applications for the objective pursued and the type of digital content to optimize creations.	Case 1: Question 7
**3.2 Designing, integrating, and reworking digital content in various formats**		
	The respondent creates and publishes complex content suited to the audiences, objectives, or purposes thereof, seeking the most appropriate content in each case (using a template).	Case 1: Question 3
	The respondent uses and evaluates repositories of audiovisual content (images, audio recordings, GIFs^a^, videos, templates, etc) to design or rework digital content.	Case 2: Question 5
**4.1 Protecting digital systems, devices, and content**		
	The respondent protects digital files and content to prevent unauthorized access by third parties.	Case 2: Question 9
**4.2 Protecting personal data and privacy**		
	The respondent identifies critical points and suggests improvements for protecting personal data and privacy. Applying best practices, the respondent customizes the privacy settings of digital tools and environments and the permissions of apps to protect their identity and the privacy of the content they generate.	Case 2: Question 10
**4.3 Acting in a civic manner in the digital environment**		
	The respondent uses licenses and attribution systems suited to their objectives when publishing content in digital environments.	Case 2: Question 2
	The respondent promotes coexistence in the digital environment.	Case 2: Question 8
**5.1 Understanding the basics and using digital technology**		
	The respondent uses digital technology and its environment autonomously.	Case 2: Question 11
**5.2 Identifying personal and professional needs and applying digital solutions**		
	The respondent is up to date with the latest technological trends and compares and evaluates digital devices and tools to select those that best meet their personal needs (leisure, health, protection, sports, emotional, etc) and professional needs (training, job search, productivity, and time management).	Case 2: Question 6

^a^GIF: Graphics Interchange Format.

### Data Collection

The survey was created and distributed to HPs using Microsoft Forms, together with an invitation email presenting the objective and characteristics of the study. Several meetings were held with HP associations and health service providers to explain our intention to conduct this study and that we would need their help to reach HPs. The invitation was distributed through the Catalan Department of Health and the Catalan Health Service, which sent it to the corresponding professional associations and to the human resources departments of public and private health care providers (hospitals, consortia, etc). Each institution decided how to disseminate the study among the professionals.

Before participating in the study, the participants had to provide consent for the study sponsor to process the information collected. The time taken to complete the survey (in minutes) was recorded.

### Sample Size

The sample size was calculated so that it would be representative of the population of Catalan HPs (both in size and distribution). Of a total of 121,039 HPs [30], 36,520 (30.17%) were physicians, 45,995 (38%) were nurses, and 38,524 (31.83%) were other HPs. In 2019, 96,105/121,039 (79.40%) and 11,014/121,039 (9.10%) of these HPs worked in the Barcelona and Girona health care regions, respectively. The remaining (13,920/121,039, 11.50%) worked in other health care regions [14,30]. The minimum and maximum sample sizes were calculated using the Cochran formula, considering a 95% power and a 10% and 5% margin of error, respectively. This resulted in a sample size of 304 to 906 and 34 to 101 HPs in the Barcelona and Girona health care regions, respectively, and 45 to 134 HPs in other health care regions. The minimum and maximum overall sample sizes were in the range of 383 to 1141 HPs.

### Statistical Analysis

Percentages and mean (SD) were used to summarize categorical and continuous variables, respectively. Categorical variables were compared using the chi-square test, whereas continuous variables were compared using the *t* test or ANOVA (for 2 or >2 comparators, respectively). These comparisons were 2-sided. The analysis of subgroups with a score of <25 in the digital competence test was performed using a 1-sided *t* test. Given that no maximum time to complete the survey was established and that the filling in of fields could be interrupted for personal or professional reasons, atypical observations where this was likely to have happened were removed to estimate a more realistic mean completion time. Outliers were filtered using the Hampel identifier. The internal consistency of the survey was analyzed using the greatest lower bound, given the lack of homogeneity of the scoring scale. The discriminatory index of each question of the ACTIC-derived digital competence test was calculated according to the study by Taib et al [18], where a score of ≥0.2 indicated good discrimination between HPs with intermediate and basic levels. Statistical analyses were performed using the R (version 4.11; R Foundation for Statistical Computing) software. A *P* value of <.05 was considered significant.

### Ethics Approval

No ethics approval was required due to the type and nature of the study as the Catalan Department of Health is responsible for formulating the general criteria for health planning, setting the objectives, and the levels to be achieved in the topics that are included in the Health Plan for Catalonia [31]. All participants were informed about the study’s purposes and that their participation was voluntary. Data protection treatment was informed to the participant and before accessing the survey, participants had to provide acceptance.

## Results

Between June 28 and July 16, 2021, a total of 1009 potential participants accessed the survey, of whom 922 (91.40%) gave their consent to participating in it. Of these, 803 (79.6%) participants were classified as HPs according to the legal definition and constituted the study population.

### Descriptive Analysis

Table 2 shows the demographic and professional characteristics of the participants, most of whom were women (612/803, 76.2%), aged between 36 and 55 years (438/803, 54.5%). Nursing was the most common professional profile (227/803, 28.3%), followed by physicians (176/803, 21.9%). Nearly half of the participants worked in a subsidized private center. A total of (478/803, 59.5%) participants worked in specialized health care settings. Barcelona city was the most common work setting (209/803, 26%), followed by Camp de Tarragona (148/803, 18.4%). The mean length of professional experience was 19.6 (SD 11) years.

**Table 2:** Demographic and professional characteristics of participants (N=803).

Variables		Values
**Gender, n (%)**		
	Women	612 (76.2)
	Men	188 (23.4)
	Nonbinary	3 (0.4)
**Age (years), n (%)**		
	18-25	24 (3)
	26-35	169 (21)
	36-45	258 (32.1)
	46-55	180 (22.4)
	56-65	155 (19.3)
	>65	17 (2.1)
**Health care professional profiles, n (%)**		
	Nurse	227 (28.3)
	Physician	176 (21.9)
	Physiotherapist	80 (10)
	Occupational therapist	74 (9.2)
	Podiatrist	58 (7.2)
	Dietitian-nutritionist	49 (6.1)
	Speech therapist	38 (4.7)
	Psychologist	35 (4.4)
	Pharmacist	32 (4)
	Biologist	15 (1.9)
	Dental hygienist	12 (1.5)
	Physicist or chemist	3 (0.4)
	Optician-optometrist	2 (0.2)
	Dentist	1 (0.1)
	Dental technician	1 (0.1)
**Workplace ownership, n (%)**		
	Public	257 (32)
	Private (subsidized)	365 (45.5)
	Private (nonsubsidized)	170 (21.2)
	Do not know or no answer	11 (1.4)
**Level of health care,^a^ n (%)**		
	Mental health and addictions	67 (8.3)
	Hospital or specialized care	478 (59.5)
	Primary care	199 (24.8)
	Social health	125 (15.6)
**Work setting,^b^ n (%)**		
	Alt Pirineu i Aran	26 (3.2)
	Barcelona city	209 (26)
	Camp de Tarragona	148 (18.4)
	Catalunya Central	59 (7.3)
	Girona	67 (8.3)
	Lleida	42 (5.2)
	Metropolitan (north)	119 (14.8)
	Metropolitan (south)	73 (9)
	Terres de l’Ebre	53 (6.6)
	Do not know or no answer	7 (0.9)
Professional experience (years), mean (SD)		19.6 (11)

^a^Multiple responses were allowed.

^b^Catalan health regions.

Table 3 shows the information collected on digital competence and on the use of, training needs for, and interest in digital tools. Most participants self-rated their digital competence as intermediate (384/803, 47.8%) or basic (357/803, 44.5%). Nearly half of the participants (394/803, 49.1%) did not know about ACTIC certification. Office tools (Microsoft Office, email, etc) were the most frequently used professional digital tools (750/803, 93.4%), followed by social media (700/803, 87.1%) and electronic health records (574/803, 71.5%). The most in-demand training topics were tools for disease prevention and health promotion (383/803, 47.7%), office tools (358/803, 44.6%), electronic health records (346/803, 43.1%), remote follow-up tools (300/803, 37.4%), and decision-making support tools (247/803, 30.8%). In relation to the use of digital tools for professional purposes and training needs, expressed for the largest groups of HPs in this study, we found that the tools that nurses (227/803, 28.3%) used the most (>30%) were office tools (206/227, 90.7%), social networks (198/227, 87.2%), electronic health records (192/227, 84.6%), healing support tools (83/227, 36.6%) and health promotion tools (80/227, 35.2%). Of these, the ones with more training needs were health promotion tools (106/227, 46.7%), office tools (94/227, 41.4%), electronic health records (93/227, 41%), healing support tools (91/227, 40.1%), and social networks (41/227, 18.1%). For physicians (176/803, 21.9%), we found that office tools (169/176, 96%), electronic health records (161/176, 91.5%), social networks (154/176, 87.5%), prescription tools (152/176, 86.4%), remote follow-up tools (68/176, 38.6%), epidemiological register tools (63/176, 35.8%), decision-making support tools (58/176, 33%), and health promotion tools (55/176, 31.3%) were the most used tools. Of these, the ones with more training needs were decision-marking support tools (81/176, 46%), office tools (78/176, 44.3%), electronic health records (77/176, 43.8%), remote follow-up tools (74/176, 42%), health promotion tools (69/176, 39%), prescription tools (58/176, 33%), epidemiological register tools (48/176, 27.3%), and social networks (40/176, 22.7%). As for physiotherapists (80/803, 10%), the most used tools were office tools (74/80, 93%), social networks (69/80, 86%), electronic health records (49/80, 61%), and health promotion tools (31/80, 39%), whereas health promotion tools (45/80, 39%), office tools (37/80, 46%), electronic health records (37/80, 46%), and social networks (36/80, 45%) were the most in-demand training topics. For occupational therapists (74/803, 9.2%), we found that (72/74, 97%), social networks (59/74, 80%), and electronic health records (49/74, 66%) were the most used tools, whereas office tools (30/74, 41%), electronic health records (30/74, 41%), and social networks (18/74, 24%) were the most in-demand training topics. As for podiatrists (58/803, 7.2%), the most used tools were social networks (53/58, 91%), office tools (51/58, 88%), electronic health records (29/58, 50%), and prescription tools (19/58, 33%), whereas electronic health records (25/58, 43%), prescription tools (20/58, 35%), office tools (16/58, 28%), and social networks (15/58, 26%) were the most in-demand training topics. Moreover, for dietitian-nutritionists (49/803, 6.1%), we found that social networks (46/49, 94%), office tools (44/49, 90%), electronic health records (26/49, 53%), health promotion tools (21/49, 43%), and remote follow-up tools (17/49, 35%) were the most used tools, whereas health promotion tools (34/49, 69%), electronic health records (24/49, 49%), office tools (23/49, 47%), and social networks (20/49, 41%) were the most in-demand training topics.

Disaggregated information relating to “Others” participants (biologists and dietitians or nutritionists, pharmacists, physiotherapists, dental hygienists, speech therapists, podiatrists, psychologists, and occupational therapists) can be found in Multimedia Appendix 2.

The greatest interest in receiving training in digital tools for personal purposes was for presentation of digital content (426/803, 53.1%); data management (408/803, 50.1%); digital technology, computer, and operating system use (344/803, 42.8%); and processing of graphic, audio, and video information (341/803, 42.5%; Table 3).

**Table 3:** Participants’ digital competences and use of, training needs for, and interest in digital tools (N=803).

Variables		Overall, n (%)	Physicians, n (%)	Nurses, n (%)	Others, n (%)
**Self-perceived digital competence**					
	Advanced	54 (6.7)	14 (8)	14 (6.2)	26 (6.5)
	Intermediate	384 (47.8)	80 (45.5)	104 (45.8)	200 (50)
	Basic	357 (44.5)	81 (46)	107 (47.1)	169 (42.3)
	No digital competence	8 (0.1)	1 (0.6)	2 (0.9)	5 (1.3)
**Accreditation of Competence in Information and Communication Technologies-2 certificate or similar**					
	Yes	57 (7.1)	3 (1.7)	32 (14.1)	22 (5.5)
	No	352 (43.8)	70 (39.8)	109 (48)	173 (42.3)
	I do not know about the ACTIC certificate	394 (49.1)	103 (58.5)	86 (37.9)	205 (51.3)
**Use of digital tools for professional purposes^a,b^**					
	Office tools (Microsoft Office, email, etc)	750 (93.4)	169 (96)	206 (90.7)	372 (93)
	Social media	700 (87.1)	154 (87.5)	198 (87.2)	348 (87)
	Electronic health records	574 (71.5)	161 (91.5)	192 (84.6)	218 (54.5)
	Prescription tools	265 (33)	152 (86.4)	53 (23.3)	60 (15)
	Health promotion tools	246 (30.6)	55 (31.3)	80 (35.2)	111 (27.8)
	Remote follow-up of patients	219 (27.3)	68 (38.6)	50 (22)	101 (25.3)
	Decision-making support tools	159 (19.8)	58 (33)	60 (26.4)	41 (10.3)
**Training needs for professional purposes^a,b^**					
	Health promotion tools	383 (47.7)	69 (39.2)	106 (46.7)	205 (51.3)
	Office tools (Microsoft Office, email, etc)	358 (44.6)	78 (44.3)	94 (41.4)	181 (45.3)
	Electronic health records	346 (43.1)	77 (43.8)	93 (41)	172 (43)
	Remote follow-up of patients	300 (37.4)	74 (42)	67 (29.5)	156 (39)
	Decision-making support tools	247 (30.8)	81 (46)	61 (26.9)	102 (25.5)
	Prescription tools	215 (26.8)	58 (33)	82 (36.1)	72 (18)
	Social networks	212 (26.4)	40 (22.7)	41 (18.1)	132 (33)
	Diagnostic support tools	196 (24.4)	67 (38.1)	37 (16.3)	89 (22.3)
	Bioinformatic (Omics) tools	172 (21.4)	55 (31.3)	49 (21.6)	65 (16.3)
	Epidemiological register tools	141 (17.6)	48 (27.3)	53 (23.3)	54 (13.5)
	Healing support tools	158 (19.7)	18 (10.2)	91 (40.1)	29 (7.3)
**Personal interest in digital training^a,b^**					
	Digital content presentation	426 (53.1)	81 (46)	126 (55.5)	219 (54.8)
	Data management	408 (50.1)	97 (55.1)	113 (49.8)	198 (49.5)
	Digital technology, computer, and operating system use	344 (42.8)	75 (42.6)	113 (49.8)	156 (39)
	Web browsing and digital communication	344 (42.8)	78 (44.3)	105 (46.3)	161 (40.3)
	Graphic, audio, and video information management	341 (42.5)	82 (46.6)	99 (43.6)	160 (40)
	Written information management	301 (37.5)	59 (33.5)	88 (38.8)	154 (38.5)
	Numeric information management	235 (29.3)	57 (32.4)	72 (31.7)	106 (26.5)
	Culture, participation, and citizenship	244 (27.9)	40 (22.7)	70 (30.8)	114 (28.5)

^a^Multiple responses were allowed.

^b^Only those options to which >15% of participants responded are shown.

### Digital Competence Test

**Figure 1:**
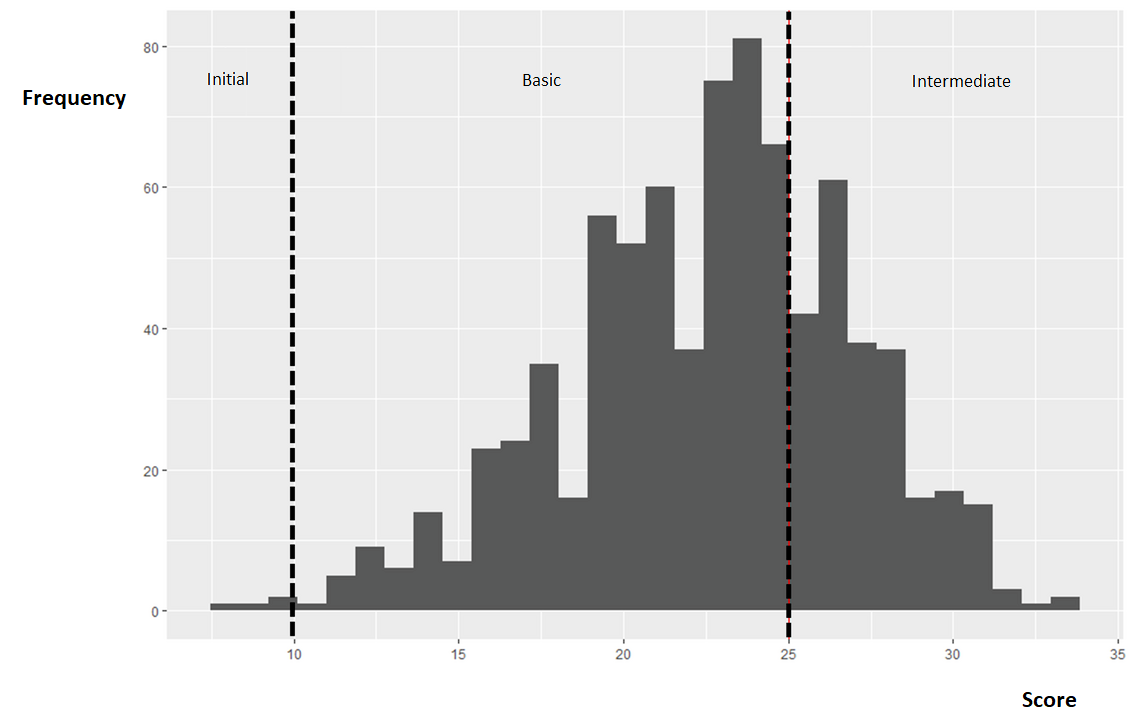
Distribution of scores achieved in the digital competence test.

The mean score in the digital competence test was 22.6 (SD 4.3), so most participants displayed a basic level (Table 4 and Figure 1). After removing outliers (122/803, 15%; see the *Methods* section), the mean time taken to complete the survey was 15.7 (SD 5.8) minutes.

**Table 4:** Digital competence test results (N=803).

Variable		Overall population	Accreditation of Competence in Information and Communication Technologies-2 certificate holders (n=57)
Score, mean (SD)		22.6 (4.3)	23.4 (3.9)
**Score range, n (%)**			
	<10 (initial level)	2 (0.2)	0 (0)
	10-24.9 (basic level)	535 (66.7)	34 (59.6)
	≥25 (intermediate level)	266 (33.1)	23 (40.4)
Time to complete the survey, (minutes), mean (SD)		15.7 (5.8)	11.8 (3.6)

The test score was higher in men (mean 23.2, SD 4.2, vs mean 22.4, SD 4.3 in women; *P*=.03), in younger participants (*P*<.001), and those with a high self-perceived level of digital competence (*P*<.001). We also observed differences among the various HP profiles, with other HP profiles scoring higher than nurses and physicians. Most of the analyzed subgroups had scores that were significantly <25 (Table 5).

A multivariate linear regression analysis confirmed the significant differences found in age, HP profile, and self-perception, but this was not confirmed for the gender variable.

In total, 7.1% (57/803) of participants had the ACTIC-2 certificate. Their mean score in the digital competence test was 23.4, SD 3.9), with 59.6% (34/57) having a basic level. The mean time taken to complete the survey was 11.8 (SD 3.6) minutes (Table 4). Of those 57 participants, 13 (22.8%) said their self-perceived knowledge was advanced, and 33 (57.9%) said their self-perceived knowledge was intermediate.

The internal consistency of the digital competence test as measured by greatest lower bound was 0.66 (acceptable consistency). The discrimination index of all questions except for one for all items was ≥0.2.

**Table 5:** Scores achieved in the digital competence test according to participants’ characteristics (N=803)^a^.

Characteristics				Value, n (%)	Score, mean (SD)			*P* value						Intermediate level achievement, *P* value	
**Gender**									.03						
	Women			612 (76.2)	22.4 (4.3)									<.001	
	Men			188 (23.4)	23.2 (4.2)									<.001	
**Age (years)**									<.001						
	18-25			24 (3)	24.6 (3.2)									.28	
	26-35			169 (21.1)	23.5 (4)									<.001	
	36-45			258 (32.1)	22.6 (4.7)									<.001	
	46-55			180 (22.4)	22.5 (4.1)									<.001	
	56-65			155 (19.3)	21.6 (4.1)									<.001	
	>65			17 (2.1)	21.5 (4.9)									.004	
**Health care professional profiles**									<.001						
		Nurse		227 (28.3)			21.7 (4.2)							<.001	
		Physician		176 (21.9)			22.5 (4.2)							<.001	
		Other		400 (49.8)			23.1 (4.4)							<.001	
		Biologist		15 (1.9)			23.3 (5.1)							.10	
		Dietitian-nutritionist		49 (6.1)			24 (4.3)							.05	
		Pharmacist		32 (4)			23.6 (4.9)							.06	
		Physiotherapist		80 (10)			22.6 (4)							<.001	
		Dental hygienist		12 (1.5)			20 (5)							.003	
		Speech therapist		38 (4.7)			24.6 (3.9)							.24	
		Podiatrist		58 (7.2)			22.3 (4.6)							<.001	
		Psychologist		35 (4.4)			23.8 (5.1)							.09	
		Occupational therapist		74 (9.2)			23 (4)							<.001	
**Workplace ownership**												.40			
		Public		257 (32)	22.5 (4.4)								<.001		
		Private (subsidized)		365 (45.5)	22.5 (4.3)								<.001		
		Private (nonsubsidized)		170 (21.2)	23.1 (4.3)								<.001		
**Level of health care**												.24			
		Mental health and addictions		67 (8.3)	23.5 (3.9)								.001		
		Hospital or specialized care		478 (59.5)	22.5 (4.4)								<.001		
		Primary care		199 (24.8)	22.5 (4.3)								<.001		
		Social health		125 (15.6)	23 (4.4)								<.001		
**Work setting**											.08				
		Alt Pirineu i Aran		26 (3.2)	21.8 (2.9)								<.001		
		Barcelona city		209 (26)	23.5 (4.3)								<.001		
		Camp de Tarragona		148 (18.4)	22 (4.2)								<.001		
		Catalunya Central		59 (7.4)	22.8 (4.5)								<.001		
		Girona		67 (8.3)	22.3 (4.5)								<.001		
		Lleida		42 (5.2)	21.7 (4.4)								<.001		
		Metropolitan (north)		119 (14.8)	22.6 (4.2)								<.001		
		Metropolitan (south)		73 (9.1)	23 (5.1)								<.001		
		Terres de l’Ebre		53 (6.6)	22 (3.9)								<.001		
**Self-perceived digital competence**												<.001			
			Advanced	54 (6.7)	24.6 (3.8)								.21		
			Intermediate	384 (47.8)	23.6 (3.9)								<.001		
			Basic	357 (44.5)	21.4 (4.3)								<.001		
			No digital competence	8 (1)	16.1 (6.3)								.002		
**Accreditation of Competence in Information and Communication Technologies-2 certificate or similar**												.11			
			Yes	57 (7.1)	23.4 (3.9)								.002		
			No, I do not know about the Accreditation of Competence in Information and Communication Technologies certificate	746 (92.9)	22.5 (4.4)								<.001		

^a^Small samples were not analyzed.

## Discussion

### Principal Findings

Globally, there is a need to improve the digital skills of HPs through dedicated training to fully exploit the potential of digital technologies and to be able to provide the best possible care using such technologies [3,11,32-34]. Multiple initiatives have been implemented to address this need [35]. For example, the EU*US eHealth Work Project devoted its efforts to identifying gaps and defining competences and developed a free introductory web-based course in eHealth [36]. Another major effort is that of Health Education England, which had defined a digital capability framework for improving the digital literacy of the health and care workforce and is currently testing a tool to self-assess digital literacy [37].

Our exploratory study in a large cohort representative of the Catalan HP population has provided valuable information regarding their digital competence level and training needs and revealed the consistency of the ad hoc digital competence test for the pursued objective. Nearly all participants (801/803, 99.8%) had either the basic or the intermediate level of competence, indicating that this HP group is already fulfilling the objectives set by the European Skills Agenda [38], whose aim is to ensure that at least 70% of adults have basic digital skills by 2025. However, the results should be interpreted with caution given the voluntary nature of participation, which might have given rise to a bias in assessing the participants’ digital competence.

Some of the findings deserve further discussion. We only found statistically significant differences in the level of digital competence by age (higher in younger ages), self-rated digital competence (higher in those rated as advanced), and professional profile (higher in HP profiles other than nurses and physicians). Conversely, we found no differences based on workplace ownership (public vs private), level of health care, or work setting, which points to a homogeneous population in this sense. Most of the analyzed subgroups obtained an overall score that was statistically <25 (basic level). Exceptions to this included the youngest participants (aged 18-25 years); those with an advanced level of self-reported digital competence; and certain HP profiles such as biologists, dietitians or nutritionists, pharmacists, speech therapists, and clinical psychologists. Although it cannot be ruled out that these professional profiles may be more highly trained in digital competences as a result of their profession, the small size of these populations may have contributed to a biased result.

Our study also provides valuable information on the most frequently used professional digital tools (Office tools, social media, and electronic health records) and the most requested type of training (tools for disease prevention and health promotion, Office tools, electronic health records, and remote follow-up tools). The training needs were found to be broader than the nature of the main tools used, and these needs were inversely proportional to the frequency of use of specific digital tools. Both findings are indicative of HPs’ interest in digital competence, which goes beyond the limitations imposed by their current professional digital skills or even by their current clinical practice. The results of an in-depth analysis of the most frequent HP profiles revealed interesting differences in the use of digital tools and training needs, with physicians displaying a broader range in terms of digital tools used and training needs [39].

Interestingly, only 7.1% (57/803) of the participants held an ACTIC-2 certificate, accrediting an intermediate level. This finding has several possible interpretations. On the one hand, the ACTIC certificate is voluntary and was conceived as a standard tool to prove citizens’ digital competences when applying for a job or job promotion. This may explain its reduced representation in our sample and the fact that nearly 50% (394/803) of the HPs participating in our study were not aware of its existence. Not holding an ACTIC-2 certificate only denotes that the participants had not had access to this voluntary certificate. This may explain the similar scores observed between the participants with or without ACTIC-2. Of the 6.7% (54/803) of participants who rated their digital competence as advanced, only 24% (13/55) had the ACTIC-2 certificate. Objectively speaking, holding this certificate translated into a higher rate of participants scoring ≥25 (intermediate level: 23/57, 40%, vs 266/803, 33.1%) and lesser time taken to complete the survey (mean 11.8, SD 3.6 minutes vs mean 15.7, SD 5.8 minutes). However, the statistical significance of these differences was not analyzed.

The findings of our study are highly valuable to the COMPDIG-Salut project as it establishes the basis for planning and deciding on specific strategic actions and policies to improve the digital competences of Catalan HPs. These are likely to be similar to other HPs in other EU countries. Furthermore, the results revealed the HPs’ training needs from both professional and personal perspectives, which may serve as a starting point for designing tailored training actions.

Comparison with similar studies is hindered by differences in the populations included, the methodology, and the questionnaires used. Further analyses through an in-depth examination of the answers obtained for each of the formulated questions are currently underway to identify specific competence areas and on which training should be primarily focused.

### Strengths and Limitations

Our study has several strengths, including a large sample size and sociodemographic representativeness of the main HP profiles in Catalonia. The distribution of the HPs’ professional profiles was skewed with respect to the real Catalan scenario, with profiles of HPs other than nurses and physicians being overrepresented.

One of the novelties of our study is the way in which digital competence is assessed, that is, subjectively by self-efficacy (the most common method) and objectively by an ad hoc survey. Although the digital competency tool is yet to be fully validated, its acceptable consistency reinforces our findings and supports its validity for use in evaluating the digital competence level in the research setting. Owing to its approach, no barriers were anticipated for its local adaptation. Moreover, all the questions answered by the participants, except one, showed sufficient discriminatory power. However, given that the tool is a generic one aimed at all HPs, the adaptation of assessment scenarios and activities to different HP profiles will enable further refinement of the results. Resolving these weaknesses in the tool will improve its accuracy.

Our study also has several limitations, some of which have already been addressed. Assuming that the gold standard of testing should be a practical test and not a survey-based case test, some of these limitations are related to the study design: these mainly include aspects related to data collection, as no information was available for the percentage of people willing to participate, the differences between respondents and nonrespondents, including how they were approached, and the response rate. Other limitations are inherent to voluntary surveys, including concerns not only about the truthfulness of the answers provided and the attention placed on certain answers but also about selection bias, as we expect HPs with higher digital skills to have been more likely to participate in the study. Differences in the interpretation of questions posed should also be considered. Finally, the exploratory nature of this study leads to hypothesis-generating conclusions rather than definitive conclusions.

### Conclusions

Knowing the digital competence level of HPs is fundamental for promoting relevant strategic policies and actions to ensure that the right resources and conditions are in place for good professional performance. Such strategies would include the design and provision of specific training to qualify and accredit the digital competences of HPs.

This exploratory study highlights the need to improve the digital competences of HPs working in Catalonia. The results have informed the Health Plan for Catalonia 2021-2025 [31] and lay the foundations for the development and deployment of a framework program for HPs’ digital competences that should include assessment indicators and standards to meet the COMPDIG-Salut project’s goals. On the basis of the definition of this digital competence framework, training methodologies and content will be developed for implementation in bachelor’s degree programs (the basic educational level for students of the various health care professions) and continuing education programs for working HPs, which in both cases must include the assessment and accreditation of digital competences.

The digital competence test showed acceptable consistency for the objective pursued, although improvements are needed to fine-tune its accuracy. The findings of this study lay the foundations for designing a strategic plan for training Catalan HPs.

## Appendix

Multimedia Appendix 1 Questionnaire on the level of digital competences of health professionals.

Multimedia Appendix 2 "Others" participants’ digital competences and use of, training needs for, and interest in digital tools.

## Abbreviations

ACTIC: Accreditation of Competence in Information and Communication Technology
COMPDIG-Salut: Digital Skills for HPs
HP: health care professional
ICT: information and communication technology

## Data Availability

We cannot disclose the entire digital competence test, as this would potentially invalidate its use in further research. An extract of the test can be found in Multimedia Appendix 1. However, the test may be made available upon request. Please contact the principal investigator for further information.

## References

[1] eHealth: Digital health and care. European Commission.

[2] Koonin LM, Hoots B, Tsang CA, Leroy Z, Farris K, Jolly B, Antall P, McCabe B, Zelis CB, Tong I, Harris AM (2020). Trends in the use of telehealth during the emergence of the COVID-19 pandemic - United States, January-March 2020. MMWR Morb Mortal Wkly Rep.

[3] Machleid F, Kaczmarczyk R, Johann D, Balčiūnas J, Atienza-Carbonell B, von Maltzahn F, Mosch L (2020). Perceptions of digital health education among European medical students: mixed methods survey. J Med Internet Res.

[4] Telemedicine: Opportunities and developments in Member State. World Health Organization.

[5] Mouratidis K, Papagiannakis A (2021). COVID-19, Internet, and mobility: the rise of telework, telehealth, e-learning, and e-shopping. Sustain Cities Soc.

[6] Sharma V, Moulton G, Ainsworth J, Augustine T (2021). Training digitally competent clinicians. BMJ.

[7] Schreiweis B, Pobiruchin M, Strotbaum V, Suleder J, Wiesner M, Bergh B (2019). Barriers and facilitators to the implementation of eHealth services: systematic literature analysis. J Med Internet Res.

[8] Saigí-Rubió F, Vidal-Alaball J, Torrent-Sellens J, Jiménez-Zarco A, López Segui F, Carrasco Hernandez M, Alzaga Reig X, Bonet Simó JM, Abizanda González M, Piera-Jimenez J, Solans O (2021). Determinants of Catalan public primary care professionals' intention to use digital clinical consultations (eConsulta) in the post-COVID-19 context: optical illusion or permanent transformation?. J Med Internet Res.

[9] Lolich L, Riccò I, Deusdad B, Timonen V (2019). Embracing technology? Health and social care professionals' attitudes to the deployment of e-Health initiatives in elder care services in Catalonia and Ireland. Technol Forecast Soc Change.

[10] Konttila J, Siira H, Kyngäs H, Lahtinen M, Elo S, Kääriäinen M, Kaakinen P, Oikarinen A, Yamakawa M, Fukui S, Utsumi M, Higami Y, Higuchi A, Mikkonen K (2019). Healthcare professionals' competence in digitalisation: a systematic review. J Clin Nurs.

[11] Socha-Dietrich K (2021). Empowering the health workforce to make the most of the digital revolution. Organisation for Economic Co-operation and Development.

[12] Pérez Sust P, Solans O, Fajardo JC, Medina Peralta M, Rodenas P, Gabaldà J, Garcia Eroles L, Comella A, Velasco Muñoz C, Sallent Ribes J, Roma Monfa R, Piera-Jimenez J (2020). Turning the crisis into an opportunity: digital health strategies deployed during the COVID-19 outbreak. JMIR Public Health Surveill.

[13] Temprana-Salvador J, López-García P, Castellví Vives J, de Haro L, Ballesta E, Rojas Abusleme M, Arrufat M, Marques F, Casas JR, Gallego C, Pons L, Mate JL, Fernández PL, López-Bonet E, Bosch R, Martínez S, Ramón Y Cajal S, Matias-Guiu X (2022). DigiPatICS: digital pathology transformation of the Catalan health institute network of 8 hospitals-planification, implementation, and preliminary results. Diagnostics (Basel).

[14] Reunió plenària I Fòrum de diàleg professional. Departament de Salut.

[15] Committee on Digital Skills for Health Professionals (2016). Digital skills for health professionals. European Health Parliament.

[16] Karnoe A, Furstrand D, Christensen KB, Norgaard O, Kayser L (2018). Assessing competencies needed to engage with digital health services: development of the eHealth literacy assessment toolkit. J Med Internet Res.

[17] Digital Skills Assessment Guidebook. DocPlayer.

[18] MyDigiSkills. Asociación de Universidades Populares de Extremadura.

[19] Skills and knowledge assessment and development framework. EU*US eHealth Work.

[20] Test Ikanos de competencias digitales - Gaitasun digitalen ikanos testa. Eusko Jaurlaritza.

[21] Skov A The Digital Competence Wheel. Center for Digital Dannelse.

[22] Kuek A, Hakkennes S (2020). Healthcare staff digital literacy levels and their attitudes towards information systems. Health Informatics J.

[23] What is ACTIC. gencat.

[24] The Digital Competence Framework. European Commission.

[25] Ley 44/2003, de 21 de noviembre, de ordenación de las profesiones sanitarias. Agencia Estatal Boletín Oficial del Estado.

[26] Kelley K, Clark B, Brown V, Sitzia J (2003). Good practice in the conduct and reporting of survey research. Int J Qual Health Care.

[27] (2021). ACTIC contents. Generalitat de Catalunya.

[28] Guitert M, Romeu T, Colas JF (2020). Basic digital competences for unemployed citizens: conceptual framework and training model. Cogent Educ.

[29] Fernández MÀ, Romeu T, Guitert M, Simón J, Ojando E (2022). Challenges of the New Accreditation in Digital Competences for Citizens (ACTIC): Update, Validation and Results. Zenodo.

[30] Padró municipal d’habitants - Població a 1 de gener. Per sexe, edat quinquennal i regions sanitàries. gencat.

[31] (2021). Pla de salut de Catalunya 2021-2025. Generalitat de Catalunya. Departament de Salut.

[32] Brunner M, McGregor D, Keep M, Janssen A, Spallek H, Quinn D, Jones A, Tseris E, Yeung W, Togher L, Solman A, Shaw T (2018). An eHealth capabilities framework for graduates and health professionals: mixed-methods study. J Med Internet Res.

[33] Keep M, Janssen A, McGregor D, Brunner M, Baysari MT, Quinn D, Shaw T (2021). Mapping eHealth education: review of eHealth content in health and medical degrees at a metropolitan tertiary institute in Australia. JMIR Med Educ.

[34] Echelard JF, Méthot F, Nguyen HA, Pomey MP (2020). Medical student training in eHealth: scoping review. JMIR Med Educ.

[35] Nazeha N, Pavagadhi D, Kyaw BM, Car J, Jimenez G, Tudor Car L (2020). A digitally competent health workforce: scoping review of educational frameworks. J Med Internet Res.

[36] EU*US eHealth Work.

[37] (2018). Digital literacy of the wider workforce. Health Education England.

[38] European Skills Agenda - Employment, Social Affairs and Inclusion. European Commission.

[39] Jones SS, Rudin RS, Perry T, Shekelle PG (2014). Health information technology: an updated systematic review with a focus on meaningful use. Ann Intern Med.

