# Supplementary material for: Measuring the Digital Skills of Catalan Health Care Professionals as a Key Step Toward a Strategic Training Plan: Digital Competence Test Validation Study
Source: J Med Internet Res. 2022 Nov 30;24(11):e38347. doi: 10.2196/38347 (PMC9752462; doi:10.2196/38347)
Supplement: Multimedia Appendix 1 [file jmir_v24i11e38347_app1.docx]

# Annex 1.-Questionnaire on the level of digital competences of health professionals

**Descriptive questionnaire**

1. **Gender:**

- Woman
- Man
- Non-binary

1. **Age range:**

- 18 to 25 years of age
- 26 to 35 years of age
- 36 to 45 years of age
- 46 to 55 years of age
- 56 to 65 years of age
- Over 65 years of age

1. **Health professional profile:**

- Specialist biologist
- Dietitian and Nutritionist
- Pharmacist
- Specialist physicist/chemist
- Physiotherapist
- Dental hygienist
- Nurse
- Speech therapist
- Doctor
- Dentist
- Optician Optometrist
- Podiatrist
- Prosthetic Dentist
- Clinical or general health psychologist
- Occupational Therapist
- Other. Which? ________

1. **Working in a centre that is:**

- Publicly owned
- Privately owned – subsidized
- Privately owned – not subsidized
- I don't know

1. **Healthcare division in which you carry out your professional activity:**

- Primary Care
- Specialized Hospital Care
- Socio-sanitary Care
- Mental Health Care and Addictions
- Other. Which? ________

1. **You currently carry out your professional activity in the region of:**

- Alt Pirineu i Aran
- Barcelona ciutat
- Metropolitana Nord
- Metropolitana Sud
- El Camp de Tarragona
- Catalunya Central
- Girona
- Lleida
- Terres de l'Ebre
- I don't know

1. **Number of years working in the health sector___________**
2. **In relation to the use of digital technologies, what level do you consider yourself?:**

- **Basic User**: I have acquired the basic digital skills to be present and participate in the digital ecosystem. I can identify devices and technologies and know and use applications, technologies and principles (of access and use).
- **Medium user**: I have an active role in the digital ecosystem (I participate, create and disseminate content). I use the advanced features of technological tools and applications and can apply critical capacity and autonomy in the use of digital technologies.
- **Advanced User**: I have the most advanced digital skills to transform and innovate in today’s digital society. I promote digital projects and advise other people on their path to achieving digital skills.
- **No knowledge**

1. **Select the digital tools you use most often in your professional activity.**Select one or more answers.

- Bioinformatics tools (omics data)
- Laboratory management tools (LIMS, e.g. Labware, Starlims)
- Prescription tools
- Health promotion and prevention tools
- Epidemiological registration tools (e.g. notifiable diseases)
- Remote patient tracking tools
- Decision-making tools (dashboards, indicators, etc.)
- Hospital pharmacy management support tools (e.g. SILICON)
- Care support tools (e.g. GACELA)
- Operations support tools (HR, logistics, economic/financial, etc.)
- Diagnostic support tools (e.g. SIMdCAT, other RIS)
- Tools for processing official records (e.g. food products and sectors, centres, pharmacies, etc.)
- Office tools (e.g. Word, Excel, email, etc.)
- Electronic medical history (e.g. SAP, SAP-ARGOS, eCAP, HCIS, etc.)
- Social networks (e.g. WhatsApp, Telegram, Twitter, etc.)
- Other. Which? ________

1. **Specify what training you believe you need in relation to digital tools/technologies to carry out your professional activity more efficiently and/or safely?**

- Bioinformatics tools (omics data)
- Laboratory management tools (LIMS, e.g. Labware, Starlims)
- Prescription tools
- Health promotion and prevention tools
- Epidemiological registration tools (e.g. notifiable diseases)
- Remote patient tracking tools
- Decision-making tools (dashboards, indicators, etc.)
- Hospital pharmacy management support tools (e.g. SILICON)
- Care support tools (e.g. GACELA)
- Operations support tools (HR, logistics, economic/financial, etc.)
- Diagnostic support tools (e.g. SIMdCAT, other RIS)
- Tools for processing official records (e.g. food products and sectors, centres, pharmacies, etc.)
- Office tools (e.g. Word, Excel, email, etc.)
- Electronic medical history (e.g. SAP, SAP-ARGOS, eCAP, HCIS, etc.)
- Social networks (e.g. WhatsApp, Telegram, Twitter, etc.)
- Other. Which? ________

1. **On a personal level, would you like to have specific training in any of the following areas?**Select one or more answers.

- Digital culture, participation and citizenship using digital tools
- Digital technology and the use of computers and operating systems
- Browsing and communication in the digital world
- Processing of written information using digital tools
- Processing of graphics, sound and video data
- Processing of digital information using digital tools
- Data processing using digital tools
- Presentation of digital content
- Other. Which?______________

1. **Are you ACTIC 2 certified?**

- Yes
- No
- Other equivalents. Which? _________
- I don't know what it is

1. **You can add any comments in the space below**

**Digital competence test (Extract)**

**CASE 1. A new management procedure has been launched in your centre. You are asked to take part in organizing information and training sessions for your colleagues, so that you can explain it. (7 questions)**

#### To find out more about this procedure and how it has worked in other countries, you decide to search the Internet. What actions would you take at this point?

- 1. Open your browser, access a social network and search for information. After finding it, you save it to a text document.
  2. Open your browser and access a search engine. Save any interesting references to browser bookmarks.
  3. Open your browser and, through a search engine, perform searches using operators and filters. You also search for information in specific databases.
  4. Answers b and c are correct.
