# Supplementary material for: Measuring the Digital Skills of Catalan Health Care Professionals as a Key Step Toward a Strategic Training Plan: Digital Competence Test Validation Study
Source: J Med Internet Res. 2022 Nov 30;24(11):e38347. doi: 10.2196/38347 (PMC9752462; doi:10.2196/38347)
Supplement: Multimedia Appendix 2 [file jmir_v24i11e38347_app2.docx]

**Table 1.** "Others" participants’ digital competences and use of, training needs for, and interest in digital tools.

| Others | | | | | | | | | | |
| --- | --- | --- | --- | --- | --- | --- | --- | --- | --- | --- |
| Variables | N (%) | Biologists | Dietitians -Nutritionists | Pharmacists | Physiotherapists | Dental hygienists | Speech therapists | Podiatrists | Psychologists | Occupational therapists |
| **Self-perceived digital competence**, n (%) |  |  |  |  |  |  |  |  |  |  |
| Advanced | 26 (6.5) | 1 (6.7) | 2 (4.1) | 3 (9.4) | 5 (6.3) | 1 (8.3) | 5 (13.2) | 2 (3.4) | 3 (8.6) | 2 (2.7) |
| Intermediate | 200 (50.0) | 5 (33.3) | 36 (73.5) | 16 (50.0) | 36 (45.0) | 3 (25.0) | 19 (50.0) | 34 (58.6) | 10 (28.6) | 38 (51.4) |
| Basic | 169 (42.3) | 8 (53.3) | 11 (22.4) | 12 (37.5) | 39 (48.9) | 8 (66.7) | 14 (36.8) | 20 (34.5) | 21 (60.0) | 34 (45.9) |
| No digital competence | 5 (1.3) | 1 (6.7) | 0 (0.0) | 1 (3.1) | 0 (0.0) | 0 (0.0) | 0 (0.0) | 2 (3.4) | 1 (2.9) | 0 (0.0) |
| **ACTIC-2 certificate or similar**, n (%) |  |  |  |  |  |  |  |  |  |  |
| Yes | 22 (5.5) | 1 (6.7) | 5 (10.2) | 1 (3.1) | 5 (6.3) | 1 (8.3) | 0 (0.0) | 3 (5.2) | 2 (5.7) | 4 (5.4) |
| No | 173 (42.3) | 5 (33.3) | 20 (40.8) | 14 (43.8) | 36 (45.0) | 5 (41.7) | 21 (55.3) | 18 (31.0) | 13 (37.1) | 37 (50.0) |
| I don’t know the ACTIC certificate | 205 (51.3) | 9 (60.0) | 24 (49.0) | 17 (53.1) | 39 (48.8) | 6 (50.0) | 17 (44.7) | 37 (63.8) | 20 (57.1) | 33 (44.6) |
| **Use of digital tools for professional purposes^a,b^**, n (%) |  |  |  |  |  |  |  |  |  |  |
| Office tools (Microsoft office, email, etc.) | 372 (93.0) | 15 (100) | 44 (89.8) | 29 (90.6) | 74 (92.5) | 10 (83.3) | 37 (97.4) | 51 (29.1) | 33 (94.3) | 72 (97.3) |
| Social media | 348 (87.0) | 10 (66.6) | 46 (93.9) | 29 (90.6) | 69 (86.3) | 10 (83.3) | 38 (100) | 53 (30.1) | 28 (80.0) | 59 (79.7) |
| Electronic health records | 218 (54.5) | 6 (40.0) | 26 (53.1) | 9 (28.1) | 49 (61.3) | 5 (41.7) | 14 (36.8) | 29 (16.5) | 28 (80.0) | 49 (66.2) |
| Prescription tools | 60 (15.0) | 1 (6.7) | 9 (18.4) | 8 (25.0) | 11 (13.8) | 3 (25.0) | 1 (2.6) | 19 (10.8) | 0 (0.0) | 6 (8.1) |
| Health promotion tools | 111 (27.8) | 2 (13.3) | 21 (42.9) | 8 (25.0) | 31 (38.8) | 5 (41.7) | 2 (5.3) | 17 (9.7) | 8 (22.9) | 16 (21.6) |
| Remote follow-up of patients | 101 (25.3) | 4 (26.7) | 17 (34.7) | 3 (9.4) | 23 (28.8) | 1 (8.3) | 14 (36.8) | 8 (4.5) | 9 (25.7) | 21 (28.4) |
| Decision-making support tools | 41 (10.3) | 2 (13.3) | 2 (4.1) | 4 (12.5) | 11 (13.8) | 1 (8.3) | 5 (13.2) | 4 (2.3) | 1 (2.9) | 10 (13.5) |
| **Need for training for professional purposes^a,b^**, n (%) |  |  |  |  |  |  |  |  |  |  |
| Health promotion tools | 205 (51.3) | 2 (13.3) | 34 (69.4) | 16 (50.0) | 45 (56.3) | 6 (50.0) | 10 (26.3) | 32 (18.2) | 16 (45.7) | 42 (56.8) |
| Office tools (Microsoft office, email, etc.) | 181 (45.3) | 10 (66.7) | 23 (46.9) | 19 (59.4) | 37 (46.3) | 7 (58.3) | 17 (44.7) | 16 (9.1) | 19 (54.3) | 30 (40.5) |
| Electronic health records | 172 (43.0) | 8 (53.3) | 24 (49.0) | 8 (25.0) | 37 (46.3) | 5 (41.7) | 14 (36.8) | 25 (14.2) | 19 (54.3) | 30 (40.5) |
| Remote follow-up of patients | 156 (39.0) | 0 (0.0) | 22 (44.9) | 9 (28.1) | 39 (48.9) | 5 (41.7) | 11 (28.9) | 19 (10.8) | 16 (45.7) | 34 (45.9) |
| Decision-making support tools | 102 (25.5) | 5 (33.3) | 12 (24.5) | 5 (15.6) | 24 (30.0) | 4 (33.3) | 6 (15.8) | 14 (8.0) | 10 (28.6) | 21 (28.4) |
| Prescription tools | 72 (18.0) | 0 (0.0) | 7 (14.3) | 2 (6.3) | 17 (21.3) | 4 (33.3) | 2 (5.3) | 20 (11.4) | 2 (5.7) | 15 (20.3) |
| Social networks | 132 (33.0) | 4 (26.7) | 20 (40.8) | 9 (28.1) | 36 (45.0) | 4 (33.3) | 15 (39.5) | 15 (8.5) | 8 (22.9) | 18 (24.3) |
| Diagnostic supporting tools | 89 (22.3) | 2 (13.3) | 8 (16.3) | 2 (6.3) | 22 (27.5) | 4 (33.3) | 8 (21.1) | 22 (12.5) | 12 (34.3) | 7 (9.5) |
| Bioinformatic (Omics) tools | 65 (16.3) | 9 (60.0) | 6 (12.2) | 6 (18.8) | 7 (8.8) | 2 (16.7) | 3 (7.9) | 16 (9.1) | 4 (11.4) | 9 (12.2) |
| Epidemiologic register tools | 54 (13.5) | 1 (6.7) | 5 (10.2) | 5 (15.6) | 8 (10.0) | 4 (33.3) | 4 (10.5) | 15 (8.5) | 3 (8.6) | 8 (10.8) |
| Healing support tools | 29 (7.3) | 0 (0.0) | 4 (8.2) | 2 (6.3) | 4 (5.0) | 0 (0.0) | 1 (2.6) | 17 (9.7) | 0 (0.0) | 1 (1.4) |
| **Personal interest for digital training^a,b^**, n (%) |  |  |  |  |  |  |  |  |  |  |
| Digital contents presentation | 219 (54.8) | 8 (53.3) | 35 (71.4) | 18 (56.3) | 47 (58.9) | 6 (50.0) | 16 (42.1) | 26 (14.8) | 18 (51.4) | 43 (58.1) |
| Data management | 198 (49.5) | 8 (53.3) | 23 (46.9) | 21 (65.6) | 37 (46.3) | 6 (50.0) | 17 (44.7) | 26 (14.8) | 16 (45.7) | 40 (54.1) |
| Digital technology. Computer and operating system use | 156 (39.0) | 5 (33.3) | 23 (46.9) | 15 (46.9) | 32 (40.0) | 6 (50.0) | 10 (26.3) | 27 (15.3) | 10 (28.6) | 26 (35.1) |
| Web browsing and digital communication | 161 (40.3) | 3 (20.0) | 24 (49.0) | 16 (50.0) | 33 (41.3) | 9 (75.0) | 9 (23.7) | 29 (16.4) | 11 (31.4) | 23 (31.1) |
| Graphic, sound and movement image information management | 160 (40.0) | 1 (6.7) | 19 (38.8) | 10 (31.3) | 32 (40.0) | 2 (16.7) | 21 (55.3) | 28 (15.9) | 10 (28.6) | 34 (45.9) |
| Written information management | 154 (38.5) | 4 (26.7) | 21 (42.9) | 11 (34.4) | 37 (46.3) | 6 (50.0) | 17 (44.7) | 26 (14.8) | 7 (20.0) | 22 (29.7) |
| Numeric information management | 106 (26.5) | 3 (20.0) | 16 (32.7) | 10 (31.3) | 22 (27.5) | 4 (33.3) | 6 (15.8) | 17 (9.7) | 5 (14.3) | 21 (28.4) |
| Culture, participation and citizenship | 114 (28.5) | 1 (6.7) | 15 (30.6) | 10 (31.3) | 16 (20.0) | 4 (33.3) | 8 (21.1) | 19 (10.8) | 7 (20.0) | 32 (43.2) |

a: Multiple responses were allowed

b: Only those options to which >15% of participants responded are shown
